# Giuseppe Levi, Natalia Ginzburg, and *Lessico Famigliare* ( *Family Lexicon* ): a seminal figure in the history of neuron cell biology and histology seen through his daughter's literary eyes

**DOI:** 10.1055/s-0044-1786765

**Published:** 2024-05-13

**Authors:** Matheus Kahakura Franco Pedro

**Affiliations:** 1 Instituto de Neurologia de Curitiba, Departamento de Neurologia, Curitiba PR, Brazil.; 2 Instituto de Neurologia de Curitiba, Departamento de Neurorradiologia Intervencionista, Curitiba PR, Brazil.

**Keywords:** Giuseppe Levi, Natalia Ginzburg, Histology, Neurons, Giuseppe Levi, Natalia Ginzburg, Histologia, Neurônios

## Abstract

One of the most important figures in the history of neurohistology, Giuseppe Levi (1872–1965) contributed in numerous ways to neuroscience, particularly in the fields of neuronal plasticity and the understanding of sensory ganglia. His daughter Natalia Ginzburg, *née* Levi (1916–1991), on the other hand, achieved fame as one of the most celebrated Italian writers of the twentieth century. *Lessico Famigliare* ( *Family Lexicon* ), from 1963, is a semibiographical account of her life in which she describes the life and character of her father in detail, providing depth and complexity to a seminal figures in the development of neuroscience. A thorough reading of the book enables modern neurologists to fully appreciate Levi's life and contributions, by means of humanizing him and giving context to his life and works. The present article provides a summary of Levi's and Natalia's lives and times as well as an analysis of the book and of the intimate, vivid descriptions of the neurohistologist's life.

## Resumo

Uma das figuras mais importantes da história da neuro-histologia, Giuseppe Levi (1872–1965) contribuiu de diversas maneiras para a neurociência, particularmente no campo da plasticidade neuronal e na compreensão dos gânglios sensitivos. Sua filha Natalia Ginzburg, nascida Levi (1916–1991), pelo contrário, adquiriu fama como uma das escritoras italianas mais célebres do século XX. *Lessico Famigliare* ( *Léxico familiar* ), de 1963, é um relato semibiográfico de sua vida, na qual ela descreve a vida e o comportamento de seu pai em detalhes, e confere profundidade e complexidade a uma figura seminal no desenvolvimento da neurociência. Uma leitura aprofundada do livro permite que neurologistas modernos apreciem a vida e as contribuições de Levi de forma mais completa, o humanizando e dando contexto a sua vida e suas obras. O autor resume as vidas e épocas de Levi e Natalia, bem como avalia o livro e as descrições íntimas, vívidas, da vida do neurohistologista.

## INTRODUCTION

**Figure 1:**
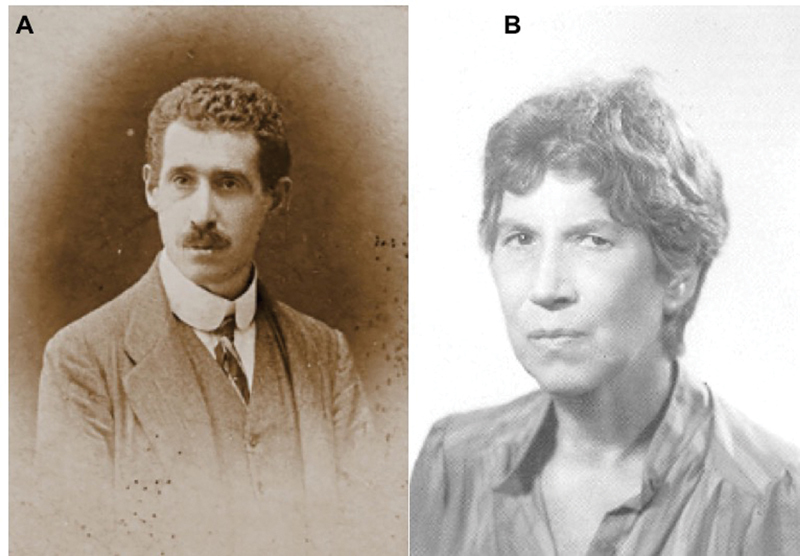
Portraits of Giuseppe Levi and Natalia Ginzburg. ( **A** ) A portrait of the neurohistologist as a young man (source: Levi-Ginzburg Archive; used with permission); ( **B** ) Natalia Ginzburg, presumably in the 1960s (source: Wikimedia Commons; public domain).

A seminal figure in neurohistology, along with Camillo Golgi (1843–1926) and Santiago Ramón y Cajal (1852–1934), 1 Giuseppe Levi (1872–1965) ( Figure 1A ) contributed in numerous ways to neuroscience. As professor at Università degli Studi di Torino, he taught a generation of Nobel Prize laureates in Physiology or Medicine. His daughter Natalia Ginzburg, *née* Levi (1916–1991) ( Figure 1B ), achieved fame as one of the most celebrated Italian authors of the twentieth century. 2 *Lessico Famigliare* ( *Family Lexicon* ), 3 from 1963, is a semibiographical account of her early life containing a multitude of details of her upbringing, emphasizing her father. It provides depth and complexity to a singular character in the development of neuroscience, enabling modern neurologists to fully appreciate Levi's life and contributions. Previous accounts on his life have dealt with *Lessico Famigliare* only tangentially; 4 5 therefore, the present article aims to provide a fuller, in-depth perspective of the book and of Levi himself.

## BIOGRAPHICAL SKETCHES

Born in Trieste, Levi graduated *cum laude* from Università degli Studi di Firenze in 1895; in Florence, he first studied neuroplasticity, 6 nuclear features of neurons, 7 8 and sensory ganglion cells. He studied briefly in Berlin, under Oskar Hertwig (1849–1922), 1 before returning to Italy. Further studies on neurons and hibernation, 9 hippocampi, 10 11 and sensory ganglia followed; he theorized on the correlation of neuronal size and an animal's size. 12 Following appointments to the universities of Sassari and Palermo, Levi earned a professorship in Turin in 1919, 1 soon gathering a remarkable group of students, including future Nobel laureates Salvador Luria (1912–1991), Renato Dulbecco (1914–2012), and Rita Levi-Montalcini (1909–2012); 1 13 despite the surnames, Levi and the latter had no familial connections. His career suffered greatly under fascism and the Second World War; he and his family first saught shelter in Liège before returning to Italy. Levi passed away in Turin, in 1965.

He and his wife, Lidia Tanzi (1878–1957), had five children; the youngest, Natalia, was born in Palermo. 2 After publishing some juvenilia, she spent most of the war following her then-husband, Leone Ginzburg (1909–1944), into exile, due to antifascist activities. Tragedy struck in 1944, as Leone was captured and brutally murdered by the Nazis. 2 14

Natalia worked at Giulio Einaudi Editore and published most of her work from the 1950s onward; 2 she became an important member of Italian literary circles, collaborating with Cesare Pavese (1908–1950). Other than *Lessico Famigliare* , which earned her the 1963 Premio Strega, 15 significant works include *Caro Michele* ( *Dear Michael* ) 16 (1973), *Famiglia* ( *Family* ) 17 (1977), and *La Famiglia Manzoni* ( *The Manzoni Family* ) 18 (1983). One of her sons, Carlo Ginzburg (1939), became a noted historian.

The aforementioned biographical data are summarized in Table 1 .

**Table 1:** Summary of the biographical aspects of Giuseppe Levi and Natalia Ginzburg

Year	1872	1895	1916	1919	1938	1939	1944	1948	1963	1965	1983	1991
Giuseppe Levi	Born in Trieste	Graduation form Università degli Studi di Firenzi		Assumes professorship in Turin		Flight from Italy due to anti-Semitic persecution		Retirement		Passes away in Turin		
Natalia Ginzburg			Born in Palermo		Marries Leone Ginzburg		Murder of Leone Ginzburg		Publishes *Lessico Famigliare* ; wins Premio Strega		Elected to the Italian Parliament as an independent candidate	Passes away in Rome

## *LESSICO FAMIGLIARE* AND A PORTRAIT OF THE FATHER

*Lessico Famigliare*^3^ is an account of Natalia's early life, family, and the words shared among them; it strongly correlates with the exceptional circumstances which her family navigated. 15 As noted by Zambra, 19 it is “the history of a Jewish, antifascist family that lives through horror, and only partially survives it”. Natalia freely admitted that, although “[she] made nothing up”, 3 “[she] wrote solely of what [she] remembered”; 3 thus, it is a memoir of the little things. 20

Her father is the dominant figure, his voice thundering in fearsome fits of temper: “anything was enough to drive him into a scary choler”; 3 “most severe in his judgments, he called everybody ‘imbecile’” 3 . Few subjects aggrieved Levi more than the fascist ascension: he “would come back furious for having found hordes of Blackshirts on his way home, or for discovering new fascists among his friends”. 3 After the family returned to Italy from Belgium, Levi faked his name, becoming “Giuseppe Lovisatto”. 3 His ethnicity led to his arrest, and he was released after “fifteen or twenty days [...] he had an overgrown beard but was proud to have been jailed”. 3

The histologist is often described as a mirror image of his beloved, more patient wife. “The things my father appreciated and held in high esteem were: socialism, England, the romances of [Émile] Zola [1840–1902], the Rockefeller Foundation, the mountain, and the guides of Val d'Aosta. The things my mother enjoyed were socialism, the poems of Paul Verlaine [1844-1896], and music, especially *Lohengrin* , which she used to sing for us, after supper”. 3 Still, conflicts with Lidia would often arise while organizing domestic *soirées* , welcoming “professors, biologists, and scientists”. 3 One of the major figures in this social environment was Tullio Terni (1888–1946), a student of Levi's, 21 admired by Natalia, who decisively introduced her to Marcel Proust's (1871–1922) œuvre. 3

Levi's habits were inflexible: “my father always got up at four in the morning”; 3 his breakfast was *mezzorado* , which he had learned to make while in Sardinia. 3 Levi dressed methodically, going to work “wearing a large cap” 3 and “a large, long coat, full of pockets and leather buttons”, 3 his hands to the back and a pipe on his lips. Financial problems plagued the family: “I don't know how we'll make do”; 3 “whenever he had to manage money, he lost it; [...] if he didn't, it was by mere chance”. 3

Levi's pleasures were few, such as “spending the summer on the mountain. We rented a house for three months, from July to September” 3 every year. Hiking and reading were his occupations; Levi resented that few of his children developed the same love for the mountains. He was fond of reading Zola and Georges Simenon (1903–1989), and enjoyed theater, having “the highest esteem” 3 for Molière (1622–1673). However, Levi “didn't just not love music, but hated it; he hated any kind of instrument that could make music, be it a piano, an accordion or a drum” 3 . Painting did not appeal to him either; he barely tolerated the old masters and took his wife to the museums begrudgingly – though “he wouldn't allow her to stop in front of a painting”; 3 modern painters, such as Felice Casorati (1883–1963), Carlo Levi (1902–1975), and Amedeo Modigliani (1884–1920) produced nothing but “smudges”. 3

In conclusion, *Lessico Famigliare* provides a broad perspective of the life of a great neurohistologist, told by a seminal writer who had been part of that life. Thus, a fuller perspective of the works and times of Giuseppe Levi enables modern neurologists to humanize him and better appreciate his contributions and his place in the development of our field.
